# *Infinity*: An In-Silico Tool for Genome-Wide Prediction of Specific DNA Matrices in miRNA Genomic Loci

**DOI:** 10.1371/journal.pone.0153658

**Published:** 2016-04-15

**Authors:** Emmanuela Falcone, Luca Grandoni, Francesca Garibaldi, Isabella Manni, Giancarlo Filligoi, Giulia Piaggio, Aymone Gurtner

**Affiliations:** 1 Department of Research, Advanced Diagnostics, and Technological Innovation, Regina Elena National Cancer Institute, Rome, Italy; 2 Department of Information Engineering, Electronics and Telecommunications (DIET), Faculty of Information Engineering, Statistics and Informatics, University Sapienza, Rome, Italy; University of Salerno, Faculty of Medicine and Surgery, ITALY

## Abstract

**Motivation:**

miRNAs are potent regulators of gene expression and modulate multiple cellular processes in physiology and pathology. Deregulation of miRNAs expression has been found in various cancer types, thus, miRNAs may be potential targets for cancer therapy. However, the mechanisms through which miRNAs are regulated in cancer remain unclear. Therefore, the identification of transcriptional factor–miRNA crosstalk is one of the most update aspects of the study of miRNAs regulation.

**Results:**

In the present study we describe the development of a fast and user-friendly software, named *infinity*, able to find the presence of DNA matrices, such as binding sequences for transcriptional factors, on ~65kb (kilobase) of 939 human miRNA genomic sequences, simultaneously. Of note, the power of this software has been validated *in vivo* by performing chromatin immunoprecipitation assays on a subset of new *in silico* identified target sequences (CCAAT) for the transcription factor NF-Y on colon cancer deregulated miRNA loci. Moreover, for the first time, we have demonstrated that NF-Y, through its CCAAT binding activity, regulates the expression of miRNA-181a, -181b, -21, -17, -130b, -301b in colon cancer cells.

**Conclusions:**

The *infinity* software that we have developed is a powerful tool to underscore new TF/miRNA regulatory networks.

**Availability and Implementation:**

*Infinity* was implemented in pure Java using Eclipse framework, and runs on Linux and MS Windows machine, with MySQL database. The software is freely available on the web at https://github.com/bio-devel/infinity. The website is implemented in JavaScript, PHP and HTML with all major browsers supported.

## Introduction

MicroRNAs (miRNAs) are a recently discovered group of small RNA molecules of about 20–25 nucleotides in length involved in the regulation of gene expression [1]. They exert their function by binding to the 3'-untranslated region of a subset of mRNAs, this results in repression of translation or directing the sequence-specific degradation of their target mRNAs. Computational predictions, supported by experimental evidences, indicate that miRNAs regulate a large fraction of metazoan genes [2,3,4,5]. In this way a large number of cellular pathways, such as cellular proliferation, differentiation and apoptosis, are regulated by miRNAs [1]. miRNA genes are generally transcribed from their own promoters by RNA Polymerase II as long primary transcripts (pri-miRNAs) often containing multiple miRNA sequences. Pri-miRNAs are cleaved into hairpin intermediates precursor (pre-miRNAs) by the nuclear RNase III Drosha and further processed to mature miRNAs by cytosolic Dicer, another RNase-III related enzyme [6,7]. Several transcription factors that regulate miRNAs transcription are strongly associated with cancer [8–10]. Thus, the deregulation of miRNAs by transcription factors may be a root cause of aberrant miRNA expression in cancer. NF-Y is an ubiquitous heterotrimeric transcription factor with a high binding affinity for the CCAAT consensus motif that is one of the most common cis-acting elements found in the promoter and enhancer regions of protein coding genes in eukaryotes in both direct (CCAAT) and reverse (ATTGG) orientation. NF-Y consists of three subunits, NF-YA, the regulatory subunit of the trimer, NF-YB, and NF-YC, all required for CCAAT binding. Growing number of experiments in cells support the notion that NF-Y complex is a key player in the regulation of proliferation and viability [11–14]. In agreement, the knock out of the NF-YA subunit in mice leads to embryo lethality [15]. Clinical studies have indicated that patients with up-regulated expression of NF-Y target genes have poor prognosis in multiple cancers [16]. NF-Y overexpression increased the proliferation rate of cancer cells harbouring endogenous mutant p53 [14]. Moreover, mutant p53 protein physically interacts with the transcription factor NF-Y up-regulating the expression of many key genes involved in the regulation of the cell cycle in response to DNA damage [17]. Analysis of transcriptome profiles during cellular transformation identified the CCAAT box as over-represented in promoters of genes overexpressed in diverse types of cancers, breast, colon, thyroid, prostate and leukemia. This strongly indicates the involvement of NF-Y in cancer-associated pathways [18]. Interestingly, computational studies indicate that a number of known transcriptional factors, among which NF-Y, could be responsible for the aberrant regulation of 214 human pre-miRNA sequences in cancer and other diseases [19]. However, no experimental data on miRNA regulation by the transcription factor NF-Y are available until now. In the present paper we describe a software, called *infinity*, with a user-friendly interface, able to find the positions of DNA matrices on 939 human miRNA genomic sequences. In particular we built 2 local databases, one with Transcription start site (TSS) annotation, genomic position, genomic annotations of human miRNA genes derived from miRStart and one containing 60kb upstream and 5kb downstream 939 pre-miRNAs, derived from Genome Browser [20]. As a proof of principle of our software, we challenged *infinity* to find NF-Y consensus sequences, CCAAT boxes, on the promoters of human miRNAs and its predictive power has been confirmed by *in vivo* chromatin immunoprecipitation (ChIPs) and loss of function experiments.

## Methods

### Data-collection

The genomic coordinates and annotations of 939 human miRNA such as name, accession number, genomic location, and strand, were uploaded in our loc l database (db) from primary database miRBase, the major available on line biological archive of miRNA sequences and annotations. TSS positions of miRNAs, cluster division, host gene and miRNA type informations were uploaded from miRStart. miRStart is an optimal resource of human miRNAs TSS because it integrates datasets from three experimental methods: (1) CAGE (Cap Analysis of Gene Expression) tags (recognize 5’-end of a gene); (2) TSS Seq tags (more than 300 million 5’-end sequences of human and mouse cDNAs by combining oligo-capping method and Solexa sequencing technology); (3) H3K4me3 enrichment surrounding TSSs [21,22]. By all these data the first table has been created. Then, we extracted from UCSC Genome Browser (grch37/hg19 (feb.2009) the sequences containing the 939 miRNAs. Since microarray profiling across 24 different human organs have demonstrated that most miRNA genes are processed from polycistronic primary transcripts [22], and, in agreement, on miRStart TSS have been analyzed in a region 50 kb upstream of each pre-miRNA [23], we decided to uploaded on our local db nucleotide strings of 60kb upstream the 5’ and 5kb downstream the 3’ of human pre-miRNAs. Furthermore, Homo sapiens genes (GRCh37) with HGNC symbols were obtained from Ensembl either to define intragenic and intergenic miRNAs or to avoid overlapping an identified TSS with other TSS of an annotated gene. Finally, a third supplementary table has been created with the FULLTEXT index [24] to take advantage of fast to search and moderate index size during the elaboration. Each database’s tables are linked with each other by logical structure based on miRNA name and accession number.

### String-matching and search examples

**Fig 1:**
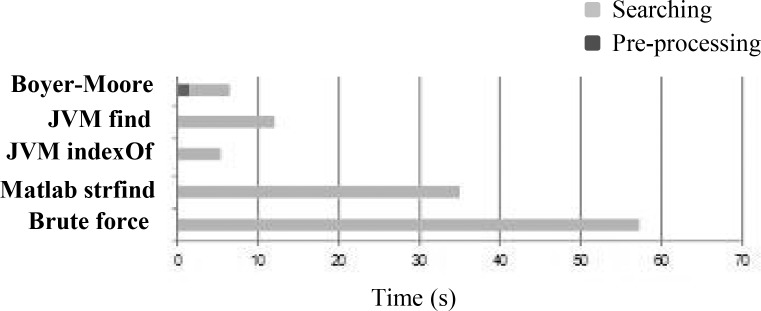
Comparison of used string matching algorithms shows the difference of elaboration time. A stream text of 65kb with 4 symbol alphabet has been processed by searching algorithm: Boyer-Moore, Brute Force, Matlab and the two algorithms provided by java, JVMfind and JVMindexOf. The pattern matrix is a 8-length characters of the same alphabet but has no match purposely. This test has been elaborated by a Sony Vaio (CPU Intel Pentium 1.10 GHz, RAM 1GB) 10,000 times to obtain the average elapsed time.

We decided to implement a relative algorithm for the exact matching in Java language because of its portable characteristics, easy user-friendly interfacing, object-oriented approach, though it lacks of a fast search algorithms (MySQL 5.5 Reference Manual. Oracle Corporation. 5 February 2013). The performances of our Java implemented algorithm have been also compared with an analogous program written in the Matlab one. In substance, the two Java commands “JVM (Java Virtual Machine) indexOF()” and “JVMfind()” implement a research method based on a “brute force” technique which compares, for each position within the text, pattern and text character identified, and returns number of occurrences and matching positions. On the other hand, the exact matching algorithm implemented by Matlab is based on the “strfind()” command that returns the starting index of each matching occurrence in a double array. If the pattern is not found or the pattern is longer than the text, the “strfind()” function returns an empty array. For our purposes, first, we compared Java (i.e., both “JVMindexOF()” and “JVMfind()”) and Matlab performances in terms of average elapsed time (AET). An exact matching test on 9 random miRNA sequences of ~65 kb was repeated one thousand times and AET was evaluated as the mean of the elapsed times in each test (Fig 1). The results have shown that Java processing is about 5 times faster than the Matlab one, and has been therefore chosen as preferential program language. If the length of nucleotide string text is not too long (i.e., less than or equal to approximately 30–40 Kb), brute-force based JAVA algorithms are preferable, otherwise we take advantage of Boyer-Moore [25] searching algorithm. Second, in order to further reduce the application execution-time, a pre-processing method was implemented directly on the database, in the SQL query. The searching procedure of the consensus matrix within the text was applied only to nucleotide sequences with almost one occurrence. This procedure used the LOCATE (substring (substr), string (str), position (pos)) (MySQL 5.5 Reference Manual. Oracle Corporation. 5 February 2013) function that returns the first occurrence of the str substr in the str, starting from position pos. If the function LOCATE (substr,str,pos) returns, no occurrence was found and miRNA will not be considered for further processing. Therefore, since the number of pattern-text matching is a-priori unknown, an optimized Java data structure of objects has been used. Each element of this structure is filled with a three-element objects array: i)-the id of the processed miRNA; ii)- the id of the pattern, derived from consensus matrix; iii)- a vector of integers containing all the occurrence indices. Finally, a user-friendly graphic interface allows users to set all the search conditions.

### Software *infinity* input window and search examples

A user-friendly graphic interface allows users to set all the search conditions. *Infinity* tool need as input a consensus matrix, limited between 4 and 30 nucleotides even setting the number and positions of possible allowable mismatches. Moreover, users need to select the DNA region of interest (all the 65kb, a region variable in length around the TSS or pre-miRNA, or import a custom sequence) and, as input, one, several or all miRNAs present in the db. Before the elaboration process, it is possible to select the type of pattern matching algorithm. At the end the results are overviewed as a table listing the number and name of miRNA founded the total number of matrices found (hits) for miRNA, the position of single hit relative to the pre-miRNA or TSS, the consensus matrix length, the elaboration time (expressed in millisecond). Furthermore, it is possible for users to find information about each miRNAs such as their accession number, genomic location, number of chromosome (ch), strand, TSS position, length, cluster (if present), host gene (if present), simply selecting the chosen table row. Finally, all results can be exported in.doc or.xls format file, or printed.

### Cell culture and infection

Human colon adenocarcinoma SW480 were grown in DMEM, supplemented with 10% FBS (GIBCO-BRL), L-glutamine (2mM), Penicillin (100 U/ml)/Streptomycin (100 ug/ml) (Life Technologies Inc.). Cells were grown at 37°C in a humidified atmosphere with 5% CO_2_. Recombinant adenoviral vector encoding dn-NF-YA-GFP (dn-NF-YA), and, as control, the adenoviral vector encoding–GFP (scr) have been described previously [14]. Cells exponentially growing on plates were infected in DMEM without serum for 1 hour at 37°C at a multiplicity of infection (MOI) of 200. Following infection DMEM supplemented with 10% FBS were added to each plate and the cells were incubate at 37°C and analyzed after 30h.

### Chromatin Immunoprecipitations (ChIP)

ChIP assays were performed in SW480 cells as described [14]. Chromatin was sonicated and incubated with 4 ug of anti-NFYA (Rockland nu200-401-100) antibody. For PCR analysis 2 ul of template in 20–30 ul of total reaction were used. PCR was performed with HOT-MASTER Taq (Eppendorf). The primers sequences of miRNA’s promoters were: 181a2-181b2-F gactagaggcagccagacac, 181a2-181b2-R cctgtctgctcagctcgcat, 21-F gggtaagaaggagctccgag, 21-R aggcacctcccactagtcag, 17-92-F cgcgcagagcttgttaacgg, 17-92-R gccccactccctcattagca, 301b-130b-F aaaccacggagcccgagact, 301b-130b-R gcgggttaaagatggagccg, 27a-F cctgaggggcacagtaaagg, 27a-R caggagaggacagaggcttc, 34a-F gggtcctgcactccaacaga, 34a-R tccttggtgccacatggacc, 183-F gctatcaccaaaccgcgagg, 183-R ggactggagcacagagacag, 31-F gggcctcagcgaggatatca, 31-R ccctaacaaggtccctaaccc, 191-F ccaagcttttcctgcccctg, 191-R ggcaacagccatttacgggc, 148a-F agaggaggtgccagctggat, 148a-R ccacacaagacccttcctgg. One of two independent experiments is represented.

### Western blotting

Cells were washed twice in ice-cold PBS, harvested by scraping with 1X RIPA buffer (150 mMNaCl, 1% TritonX100, 0.25% Sodium deoxycholate, 0.1% SDS, 50 mMTris/HClph 8.0, 20 mM EDTA) supplemented with 1X protease and phosphatase inhibitor cocktail (Sigma-Aldrich). Lysates were incubated 30 min in ice, clarified by centrifugation 20 min at 14000 RPM, and resolved onto 10% SDS-PAGE (40 ug/lane). Blotting was performed according to standard protocols and PVDF filter was immuno-reacted with following antibodies: anti-NF-YA (sc-17753, Santa Crutz Biotech.), anti-HSP70 (StressGen).

### RNA extraction, cDNA synthesis and RT-qPCR

Total RNA was extracted using the Trizol Reagent (Gibco BRL) and following the manufacturer’s instructions. Reverse Transcription of miRNAs and RT-qPCR quantification of miRNA expression were performed respectively by TaqMan MicroRNA RT assay and TaqMan MiRNA^®^ Assays (Applied Biosystems, Foster City, CA, USA) according to the manufacturer's protocol. U6 and U19 were used as endogenous controls to standardize miRNA expression. Experiments were done on triplicate and the results were estimated based on the comparative threshold (2^ΔCt^). Results are reported as fold of enrichment respect to control (scr) defined as 1.

## Results

### CCAAT-boxes are represented on human miRNA loci, both on promoter regions and intergenic regions

**Fig 2:**
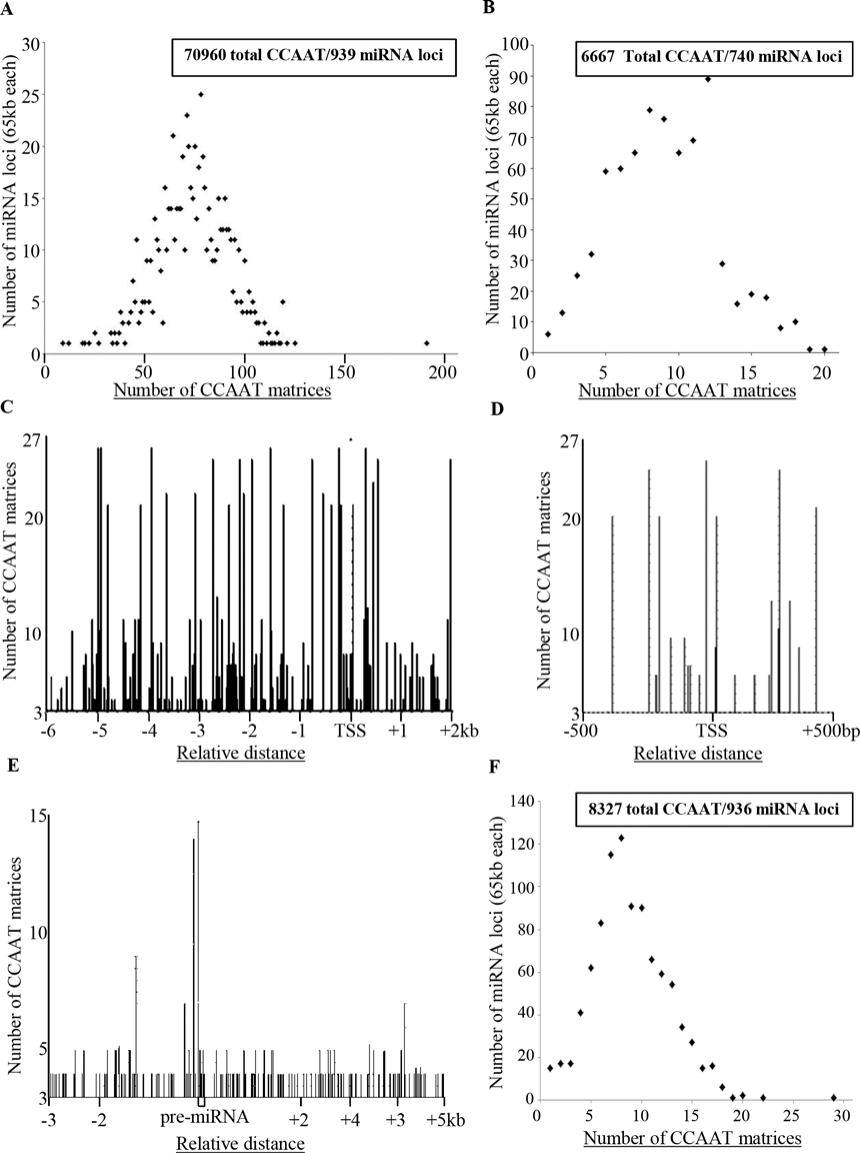
Frequency of CCAAT matrices among miRNA loci. Frequency of CCAAT matrices, found by *infinity*, (A) on 939 miRNA loci between -60kb to 5kb respect pre-miRNA; (B) on 740 miRNA loci between -6kb to 2kb respect TSS. Distribution of CCAAT matrices position, found by *infinity*, (C) on 740 miRNA loci between -6kb to 2kb respect TSS; (D) on 463 miRNA loci between -0,5kb to 0,5kb respect TSS; (E) on 936 miRNA loci between -3kb to 5kb respect pre-miRNA. (F) Frequency of CCAAT matrices, found by *infinity* on 936 miRNA loci between -3kb to 5kb respect pre-miRNA.

First, we searched for CCAAT sequences on ~65kb (60kb upstream the 5’ and 5kb downstream to the 3’of pre-miRNA) of 939 miRNA genomic loci extracted from UCSC genome browser. Since it has been demonstrated that flanking sequences of the invariably conserved CCAAT core box are important for NF-Y binding, we have searched the matrix D/V/V/C/C/A/A/T/S/N/V (D = A/T/G, N = A/T/C/G, V = C/A/G), derived from the previously described matrices [26–28]. Only matrices with no variations within the CCAAT pentanucleotide and with a match >82% in the flanking sequences were considered (CCAAT matrix). The in-silico analysis identifies a total of 70960 CCAAT matrices, in both orientations, spanning all the 939 miRNA sequences analyzed (Fig 2A, S1 Table). The number of CCAAT/ATTGG matrices identified varies from a minimum of 3 (pre-miRNA-1302-2, -1302-10) to a maximum of 160 (pre-miRNA-935) while on the 80% of miRNA genomic loci occur 50–100 CCAAT/ATTGG matrices (Fig 2A). 740 of 939 pre-miRNAs have a miRStart TSS (see Data-collection section), and we found in all of them multiple CCAAT/ATTGG matrices located on the proximal promoter region (-6kb to +2kb respect to TSS), from a minimum of 1 to a maximum of 20 CCAAT sequences on a single promoter (Fig 2B and 2C, S2 Table). Although it has been previously demonstrated that CCAAT sequences bound by NF-Y are often positioned upstream the TSS of protein coding genes between -40 to -100 bp with respect to the TSS [27, 28], we analyzed with *infinity* a minimal promoter region (-500bp to +500bp respect to the TSS). Our analysis revealed the presence of 858 CCAAT/ATTGG on 463 (62,6%) of 740 minimal promoters analyzed with an enrichment of CCAAT/ATTGG matrices spanning position between -80/-240 and +260/+450 bp, respectively. (Fig 2D, S3 Table). All together our data demonstrated that the CCAAT sequences are overrepresented on ~60% of miRNA minimal promoters and strongly support the hypothesis that NF-Y is a regulatory factor for many of these genes. Although a consistent number of data have demonstrated that NF-Y regulates transcription of genes thought its binding to CCAAT sequences on promoter and enhancer, ChIP-Seq analysis have found that NF-Y is able to binds CCAAT also on non-promoter regions as, intron, exon, 5’UTR, 3’UTR and intergenic regions [28]. In agreement, our analysis found several CCAAT/ATTGG matrices on pre-miRNA coding region and in a region close the pre-miRNAs. In particular we identified 8327 CCAAT matrices between -3kb to + 5kb respect the 5’ of 936 pre-miRNA sequences with a frequency between 1 to 22 for single miRNA (Fig 2E and 2F, S4 Table).

### *In vivo* validation of predicted NF-Y binding on miRNAs deregulated on colon cancer

Colorectal cancer (CRC) is one of the major leading causes of cancer-related death. CRC development and progression involves mutational events in oncogenes and tumor suppressor genes often accompanied by deregulated gene expression. Moreover, deregulation of miRNAs expression has been found in various cancer types among which CRC [29, 30].

Since analysis of transcriptome profiles during cellular transformation identified the CCAAT box as over-represented in promoters of genes deregulated in cancers and computational studies indicate that a number of known transcriptional factors, among which NF-Y, could be responsible for the aberrant regulation of a subset of human pre-miRNA sequences in cancer [31,20] we asked whether NF-Y binds CCAAT boxes on miRNAs loci deregulated in CRC. We first catalogued a set of common miRNAs deregulated in this tumour. In particular we searched the PubMed database to collect articles that investigated the aberrant miRNA gene expression patterns in colon samples from colon cancer patients. The keywords miRNA, microRNA and colon cancer were used in performing the literature search. This search retrieved 353 research articles until the beginning of 2013. The relevant articles extracted from PubMed were subjected to additional refinement. We selected the studies that have performed experiments on human colon cancer tissues and in autologous normal colon mucosa. Moreover, we focused our attention exclusively on up-regulated miRNAs. This search retrieved 118 up-regulated miRNAs corresponding to 141 pre-miRNAs (Table 1). All 141 sequences are enriched of CCAAT/ATTGG matrices between -3kb to + 5kb with respect the 5’ of pre-miRNA; moreover, all the 115 pre-miRNAs that have an annotated miRStart TSS, displayed multiple CCAAT/ATTGG matrices located on the promoter region (-6kb to +2kb respect to TSS), from a minimum of 1 to a maximum of 17 CCAAT/ATTGG sequences for single promoter (S5 and S6 Tables).

**Table 1:** 118 miRNA up-regulated on colorectal cancer patients. Representation of a meta-analysis on PubMed database to collect articles that investigated the aberrant miRNA gene expression patterns in colorectal samples from colorectal cancer patients. This search retrieved 118 up-regulated miRNAs in colon cancer.

miRNAs	References
miRNA-29b; miRNA-30a-5p; miRNA-140-5p; miRNA-516-3p	[36]
miRNA-99a	[36]
miRNA-15a; miRNA-27a; miRNA-98; miRNA-103; miRNA-105	[37]
miRNA-107; miRNA-122a; miRNA-128a; miRNA-134; miRNA-142-5p	[37]
miRNA-142-3p;miRNA-1; miRNA-147; miRNA-148a; miRNA-151	[37]
miRNA-181c; miRNA-186; miRNA-194; miRNA-197; miRNA-213	[37]
miRNA-215; miRNA-330; miRNA-338; miRNA-339; miRNA-370	[37]
miRNA-373; miRNA-Let-7g	[37]
miRNA-30e-3p; miRNA-181d; miRNA-220; miRNA-302a; miRNA-302b	[38]
miRNA-493-3p; miRNA-550; miRNA-570	[38]
miRNA-494; miRNA-500; miRNA-513a-5p; miRNA-513b; miRNA-513c	[39]
miRNA-892b	[39]
miRNA-337; miRNA-483-3p; miRNA-520b; miRNA-520g;	[40]
miRNA-550a-3p; miRNA-629-3p; miRNA-663; miRNA-135a-3p	[40]
miRNA-32; miRNA-33; miRNA-188; miRNA-503; miRNA-542-5p	[41]
miRNA-552; miRNA-584; miRNA-625	[41]
miRNA-451; miRNA-675	[42]
miRNA-424; miRNA-301b	[43]
miRNA-335	[44]
miRNA-10a; miRNA-25¸ miRNA-181a; miRNA-200a miRNA-200b	[36,37]
miRNA-34a; miRNA-141; miRNA-320	[36,37]
miRNA-93; miRNA-298	[36,40]
miRNA-30a-3p	[36,44]
miRNA-106b	[36,43]
miRNA-20	[46,37]
miRNA-15b; miRNA-181b	[36, 37,44]
miRNA-210; miRNA-221; miRNA-154-3p	[37,40]
miRNA-182-3p	[37,41]
miRNA-374	[37,43]
miRNA-191; miRNA-200c	[37,44]
miRNA-92a-5p	[38,40]
miRNA-196b	[38,43]
miRNA-19b	[40,42]
miRNA-203; miRNA-130b; miRNA-106	[36,37,40]
miRNA-135a	[37, 42,45]
miRNA-17-3p	[37,40,41]
miRNA-301; miRNA-92a-3p	[37,40,42]
miRNA-7	[,^38^,^39^,^40^]
miRNA-18b	[38,40,43]
miRNA-31	[36,37,38,41]
miRNA-29a	[36,37,41,42]
miRNA-95	[36,37,38,40]
miRNA-17-5p	[36,37,38,42]
miRNA-96	[36,37,40,41]
miRNA-19a	[36, 7,40,43,46]
miRNA-224	[36,37,38,41,43]
miRNA-183; miRNA-182-5p	[36,37,38,40,41]
miRNA-21	[36,37,39,40,42]
miRNA-20a	[36,37,40,42,43]
miRNA-18a	[36,38,40,42,43]
miRNA-135b	[37,40,41,43,45]

**Fig 3:**
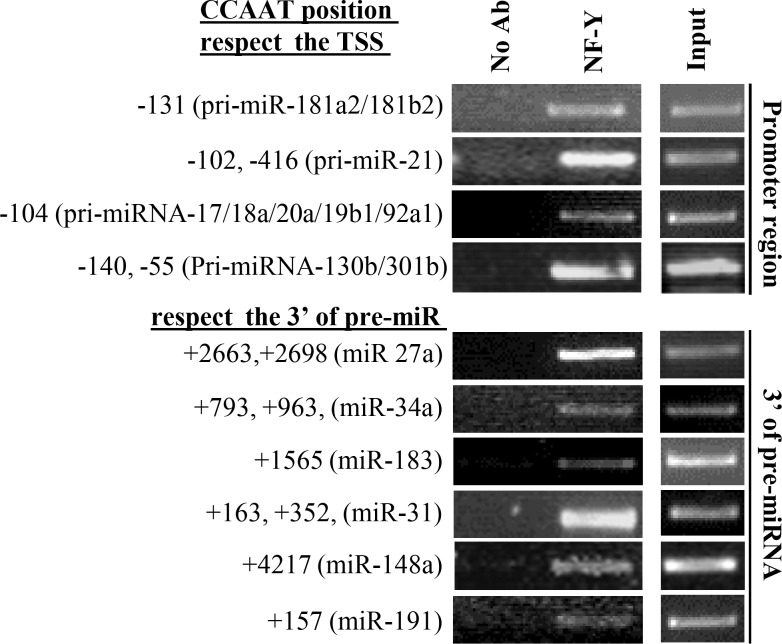
NF-Y binds *in vivo* the CCAAT-matrices on miRNAs loci. ChIPs performed on SW480 cells using the indicated antibodies. PCR were performed using specific primers for the immunoprecipitated DNA fragments (on promoter regions and downstream of 3’UTR regions) containing the indicated CCAAT-matrices found with *infinity*. Only CCAAT visualized by ChIP are indicated.

In order to demonstrate the *in vivo* binding of NF-Y to the *in silico* predicted CCAAT matrices, we concentrated our attention on promoters regions of 4 miRNA clusters (pri-miRNA-181a2/181b2, -21, -17/18a/20a/19b1/92a1, -301b/130b) coding for 10 mature miRNAs (-181a, -181b, -21, -17, -18a, -20a, -19b, -92a, -301b, -130b). Interestingly, some of them have pro-oncogenic functions on colon cancer [32–35]. These 4 promoters contain multiple CCAAT boxes (Table 2) in a region proximal to the TSS (between -500bp to -500bp). Since no data are available on miRStart for the TSS of pri-miRNA 17–92, we have considered the TSS experimentally validated by Scott M. Hammond’s group at -2918bp upstream the pre-miRNA-17 [32]. Moreover, we analyzed 6 DNA regions downstream the 3’ of pre-miRNA-27a, -34a, -183, -31, -191, -148a, containing multiple CCAAT boxes (Table 3). These miRNAs were chosen randomly. We performed *in vivo* chromatin immunoprecipitation experiments (ChIP) on human colon adenocarcinoma cells line SW480, by using anti-NF-YA antibody and, as negative controls, we included a reaction lacking primary antibody. Enrichment of DNA fragments containing predicted CCAAT/ATTGG matrices, were assessed by PCR. As shown in Fig 3, NF-Y binds *in vivo* all analyzed sequences thus strongly support the idea that *infinity* predicted CCAAT matrices are NF-Y binding consensus sites. Of note, interrogation of ChIP-seq data from 3 cell lines (ENCODE data displayed on the UCSC genome browser) corresponding to the pre-miR-181a2/181b2, -21, -17/18a/20a/19b1/92a1, -301b/130b loci reveal NF-Y binding in the same position on promoter regions that we have analyzed by ChIP. Moreover, the high levels of the activation associated histone modification H3K27ac and the RNA-seq data from ENCODE provide strong evidence that the transcription of 4 miRNA clusters is driven by putative TSS annotated miRStart (S1 and S2 Figs).

**Table 2:** Results of the *in silico* analysis of 1 kb of promoter (-0,5kb,+ 0,5kb from TSS) of the 4 miRNA clusters validated by *in vivo* experiments.

pri-miRNA	n° of CCAAT matrices	matrices	distance from TSS
mir-181a2/181b2	2	**ataattggttt**	-401
		**agcccaatcag**	**-131**
mir-21	3	**tatccaatccc**	218
		**atcccaatcat**	-416
		**ttaattggttc**	-102
mir-301b/130b	3	**cggccaatgag**	-55
		**gagattggagc**	-254
		**gggccaatcgg**	-140
mir-17/18a/20a/19b1/92a1	1	**gtgattggcgg**	-104

**Table 3:** Results of the *in silico* analysis of 5 kb downstream the 3’of the 6 pre-miRNA validated by *in vivo* experiments.

pre-miRNA	n° of CCAAT matrices	matrices	distance from 3' of pre-miRNA
pre-miRNA-27a	6	**aagccaatccc**	2698
		**ctcccaatgcg**	1657
		**aacattggtgt**	3190
		**ttgattggcct**	1981
		**ccgattggccc**	2663
		**gagccaatggt**	4899
pre-miRNA-34a	6	**tcaccaattgc**	793
		**gtgccaatatc**	3219
		**cccattggtgt**	963
		**cagccaatgcc**	3694
		**gggccaatga**	1709
		**ggaattggacc**	1321
pre-miRNA-183	4	**tgtccaatggt**	2470
		**aggccaatgtt**	769
		**gtgccaatatg**	197
		**cccccaataaa**	1565
pre-miRNA-31	9	**tgtccaataac**	2105
		**gtcattggatc**	669
		**tccattggctc**	2888
		**cacattggcct**	163
		**tttattgggaa**	705
		**aacattggtgc**	352
		**gttattggctt**	3706
		**tcaattggcca**	1714
		**taaattggggg**	3307
pre-miRNA-191	3	**Aggattggcga**	157
		**Tttattgggca**	1223
		**ggcattggccc**	3152
pre-mir-148a	5	**tgaccaattcc**	131
		**aaaattggcgc**	4593
		**tgcccaattgg**	3615
		**gagattggcag**	4217
		**ccaattggtta**	3618

### Lack of NF-Y DNA binding activity leads miRNAs down-regulation

Since some *in vivo* validated CCAAT matrices are on miRNA promoter regions, near the TSS, we hypothesized that NF-Y transcriptionally regulates these miRNAs.

**Fig 4:**
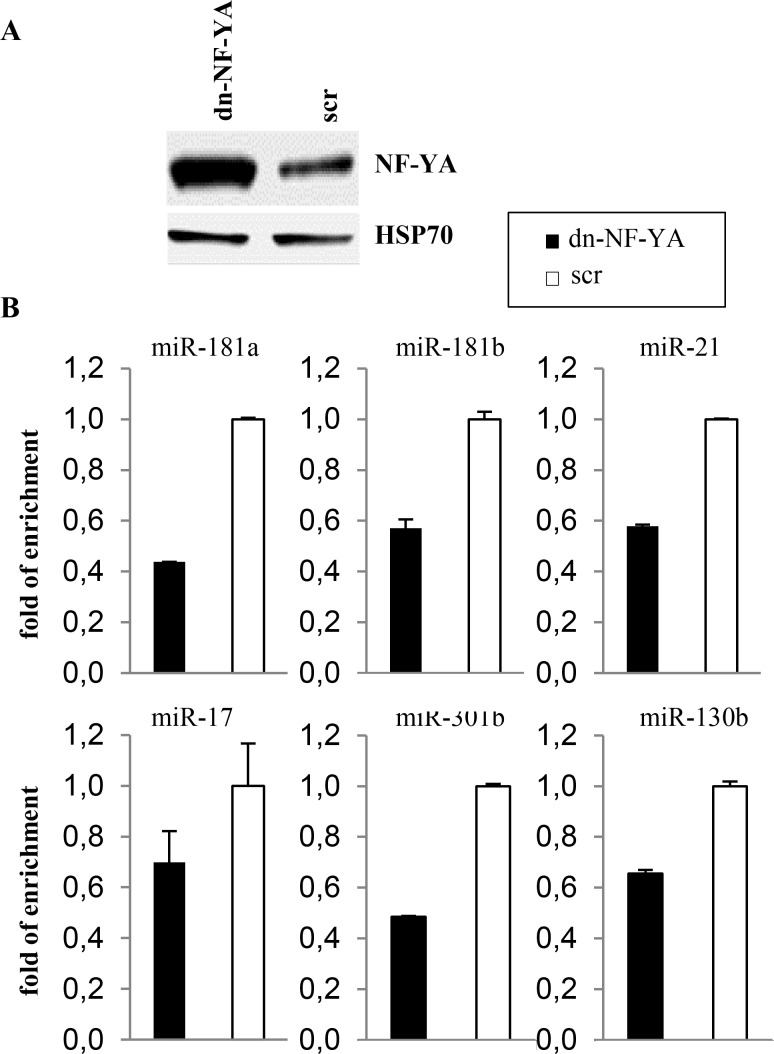
Lack of NF-Y DNA binding activity leads miRNAs down-regulation. (A) Western blot analysis performed on total lysates from colon cancer cells SW480 after 30 hours post infection with an Adenovirus-dn-NFYA-GFP (dn-NF-YA) and control Adenovirus-GFP (scr). (B) RT-qPCR analysis of the indicated mature miRNAs on RNAs from cells before and after NF-Y activity depletion by overespression of the dn-NF-Y protein. The results are shown as relative fold change of two experiments performed in triplicate.

To investigate whether NF-Y activity impacts on the expression of these miRNAs we infected SW480 cells with an adenoviral vector expressing a dominant negative NF-YA protein, dn-NF-YA (Fig 4A). This molecule is a NF-YA protein with a triple amino acid substitution in the DNA binding domain that impairs its ability to bind DNA. It is still able to interact with a NF-YB/-YC dimer, but the resulting trimer is inactive in terms of CCAAT recognition [47]. RT-qPCR analysis performed 30 hours post infection shows that upon dn-NF-YA overexpression, although to different extents, miRNA-181a, -181b, -21, -17,- 130b, -301b were down-regulated (Fig 4B).

Taken together, our data demonstrate that NF-Y binds *in vivo* a subset of miRNA’s promoters and its DNA binding activity is essential for transcription of miRNA expression in colon cancer cell line.

## Discussion

In the present study we describe the development of a fast and user-friendly software, named *infinity*. *Infinity* allows users to search a personalized consensus matrix matching simultaneously all miRNA nucleotide strings stored in our local miRNA sequence database.

To test the power of our software, we used it to search all CCAAT matrices on 60Kb upstream and 5Kb downstream of 939 human miRNA genomic sequences and we observed that CCAAT matrices are highly represented (70960 occurrences) on human miRNA loci both on promoter and non-genic regions.

It has been previously shown that CCAAT matrices are over-represented in promoters of protein coding genes deregulated in colorectal cancers and deregulation of miRNAs (non coding genes) expression has been found in colorectal cancer [18,29,30]. Thus we used our software to identify CCAAT matrices on miRNA sequences up-regulated in colon cancer and we observed that all sequences displayed multiple CCAAT/ATTGG matrices located on the 5’ and promoter regions.

The CCAAT matrix is bound *in vivo* by the NF-Y transcription factor. Of note, chromatin immunoprecipitation experiments performed with an anti-NFY antibody, strongly indicate that the *in silico* identify CCAAT matrices play an *in vivo* functional role. Indeed, NF-Y binds *in vivo* all analyzed sequences thus strongly support the idea that *infinity* predicted CCAAT matrices are NF-Y binding consensus sites. Moreover, for the first time, we have demonstrated that NF-Y, through its CCAAT binding activity, directly regulates the expression of miRNAs genes deregulated in colon cancer cells. These *in vivo* data, support a scenario in which NF-Y appears to be a regulator of gene expression regulators and, deregulating miRNAs, it may be a root cause of aberrant miRNA expression in colon cancer.

Currently, there is a plethora of available computational tools aiming to address several aspects of miRNA biology. Almost all miRNA-related researches require the extensive use of these online resources. Although there are several databases of miRNA sequences, expression, and target predictions [48], few databases on TF-miRNA interactions are available, yet. Up to day, the available tools focused on TF-miRNA research are the following. ChIPBase, TMREC and TransmiR databases that provide experimentally validated TF-miRNA interactions on diverse tissues, cell lines and diseases of different organisms [49–51]. CircuitsDB is a database devoted to the integration of transcriptional and post-transcriptional interactions (miRNA mediated), where the TF-miRNA interactions are detected by TF matrices scanning analysis on 1kb region surrounding miRNA TSS [52]. miRGen v3.0 is a database that aims to provide comprehensive information about the position of human and mouse miRNA coding transcripts and their regulation by transcription factors, including a unique compilation of both predicted and experimentally supported data [53]. The database currently supports 276 miRNA TSSs that correspond to 428 precursors. Moreover, the interconnection between miRGen v3.0 and other DIANA resources enables users to perform miRNA pathway analyses and identify miRNA predicted targets. Finally, TFmiR is a freely available web server for an integrative analysis of combinatorial regulatory interactions between TFs, miRNAs and target genes that are involved in disease pathogenesis. Finally, experimentally validated TF–miRNA interactions are integrated from TransmiR, ChIPBase and literature [54]. All these tools offer a fully functional web interface and interconnection with others miRNA-database and provide TF binding site map on miRNA promoters using DNA binding matrices for transcription factors downloaded from TRANSFAC and JASPAR or using ChIP-Seq libraries derived from ENCODE.

It is important to observe that matrices included in TRANSFAC and JASPAR do not recapitulate all TF-binding sequences and data derived from ENCODE are available only for some cell lines, thus limiting the identification of already unknown TF–miRNA networks. Moreover, some of these databases, such as CircuitsDB, have the limitation of an available short promoter region of miRNAs for the analysis. Our software allows users to search a personalized consensus matrix, in term of length and sequence, on 60Kb upstream and 5Kb downstream human miRNA sequences. This flexibility in the research offers the possibility to find not only canonical consensus for TF, but also new TF-binding sites no present in TF-databases, and also sequences with peculiar secondary structure, bound by protein complexes. This characteristics allows also to upgrade continuously a specific TF matrix (i.e. NF-Y binding matrix) based on the analysis of new in vivo experiments [18]. In summary, in our opinion, the possibility to analyze 65kb of 939 human miRNA genomic sequences simultaneously confers to infinity a useful plasticity and considerable advantages.

In the future we expect more genome-wide data to become available for human miRNAs. This will be useful to validate *infinity in-silico* data analysis. Finally, we will translate *infinity* software from java application to web interface.

## Supporting Information

S1 Table Results of in-silico analysis performed with *infinity* on 65kb of 939 human miRNA genomic loci, using the matrix D/V/V/C/C/A/A/T/S/N/V. (XLSM)

S2 Table Results of in-silico analysis performed with i*nfinity* on -6kb to +2kb with respect to the TSS of 740 human miRNA genomic loci, using the matrix D/V/V/C/C/A/A/T/S/N/V. (XLSM)

S3 Table Results of in-silico analysis performed with i*nfinity* on -500bp to +500bp with respect to the TSS of 740 human miRNA genomic loci, using the matrix D/V/V/C/C/A/A/T/S/N/V. (XLSM)

S4 Table Results of in-silico analysis performed with *infinity* on -3kb to +5kb with respect to the 5' of 939 human miRNA genomic loci, using the matrix D/V/V/C/C/A/A/T/S/N/V. (XLSM)

S5 Table Results of in-silico analysis performed with *infinity* on -6kb to +2kb with respect to the TSS of 115 human miRNA genomic loci, of wich the mature form is upregualted in colon cancer, using the matrix D/V/V/C/C/A/A/T/S/N/V. (XLSM)

S6 Table Results of in-silico analysis performed with *infinity* on -3kb to +5kb with respect to the 5' of 141 human miRNA genomic loci, of wich the mature form is upregualted in colon cancer, using the matrix D/V/V/C/C/A/A/T/S/N/V. (XLSM)

S1 Fig Screen-shot of UCSC Genome Browser at (A) miR-181a2/miR-181b2 cluster genomic locus (B) miR-21 genomic locus shows the position of mature miRNAs, transcription (RNA-seq data) and H3K27Ac ChIP-seq data (a marker of active promoter) from 9 cell lines (GM12878 (LCL), h1-hESC, HeLa-S3, HepG2, HSMM, HUVEC, K562, NHEK and NHLF), NF-Y ChIP-seq data from 3 cell lines. Arrow represent the proposed transcription start sites and the asterisk shows the position of primers used on our ChIP experiments shown on Fig 3.
(TIF)

S2 Fig Screen-shot of UCSC Genome Browser at (A) miR-17-92a cluster genomic locus (B) miR-301b/130b genomic locus shows the position of mature miRNAs, transcription (RNA-seq data) and H3K27Ac ChIP-seq data (a marker of active promoter) from 9 cell lines (GM12878 (LCL), h1-hESC, HeLa-S3, HepG2, HSMM, HUVEC, K562, NHEK and NHLF), NF-Y ChIP-seq data from 3 cell lines. Arrow represent the proposed transcription start sites and the asterisk shows the position of primers used on our ChIP experiments shown on Fig 3.
(TIF)
