# Supplementary figures and images for: Infinity: An In-Silico Tool for Genome-Wide Prediction of Specific DNA Matrices in miRNA Genomic Loci
Source: PLoS One. 2016 Apr 15;11(4):e0153658. doi: 10.1371/journal.pone.0153658 (PMC4833383; doi:10.1371/journal.pone.0153658)

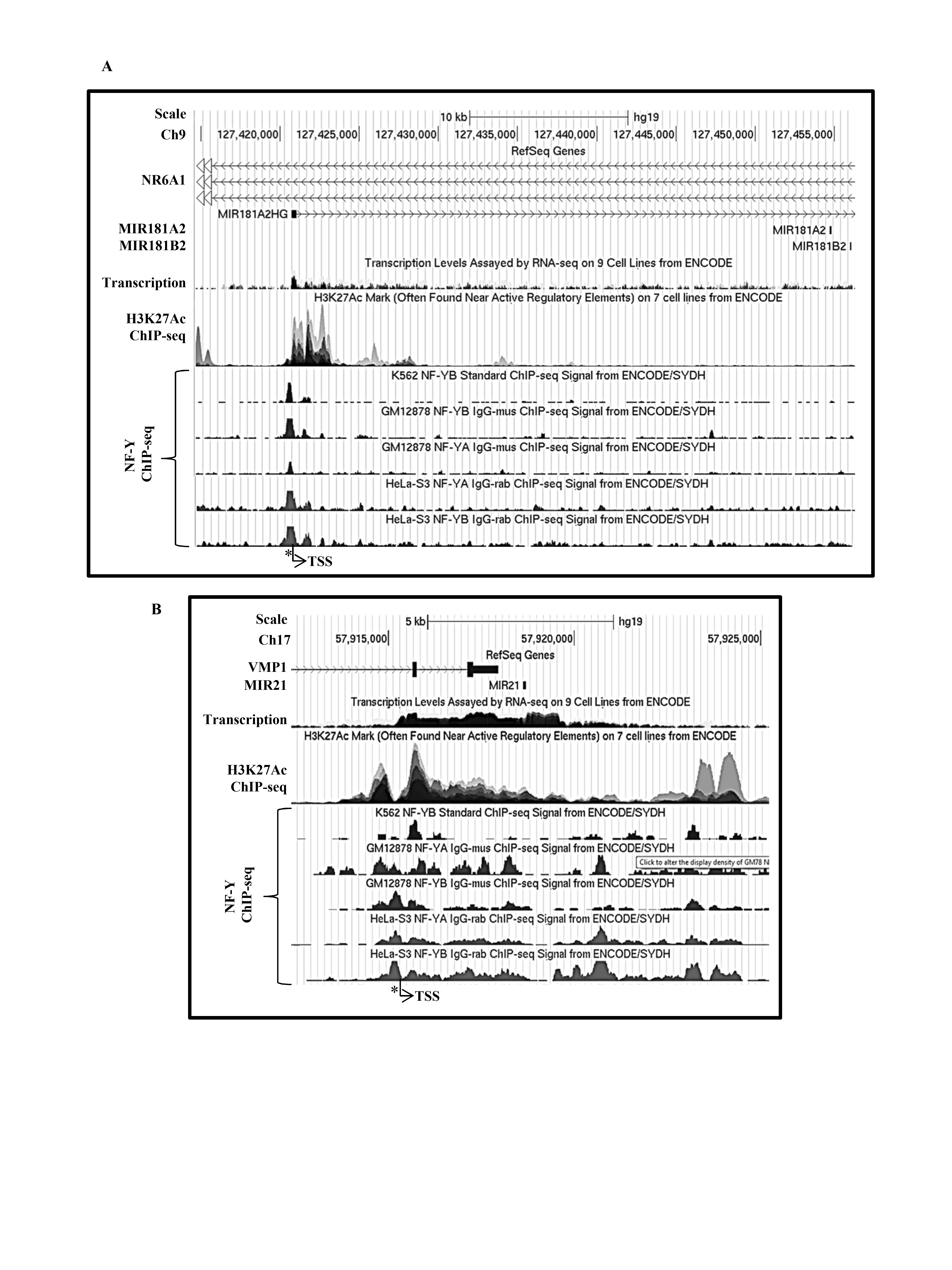

Supplement: S1 Fig — Screen-shot of UCSC Genome Browser at (A) miR-181a2/miR-181b2 cluster genomic locus (B) miR-21 genomic locus shows the position of mature miRNAs, transcription (RNA-seq data) and H3K27Ac ChIP-seq data (a marker of active promoter) from 9 cell lines (GM12878 (LCL), h1-hESC, HeLa-S3, HepG2, HSMM, HUVEC, K562, NHEK and NHLF), NF-Y ChIP-seq data from 3 cell lines. Arrow represent the proposed transcription start sites and the asterisk shows the position of primers used on our ChIP experiments shown on Fig 3. (TIF) [file pone.0153658.s007.tif]

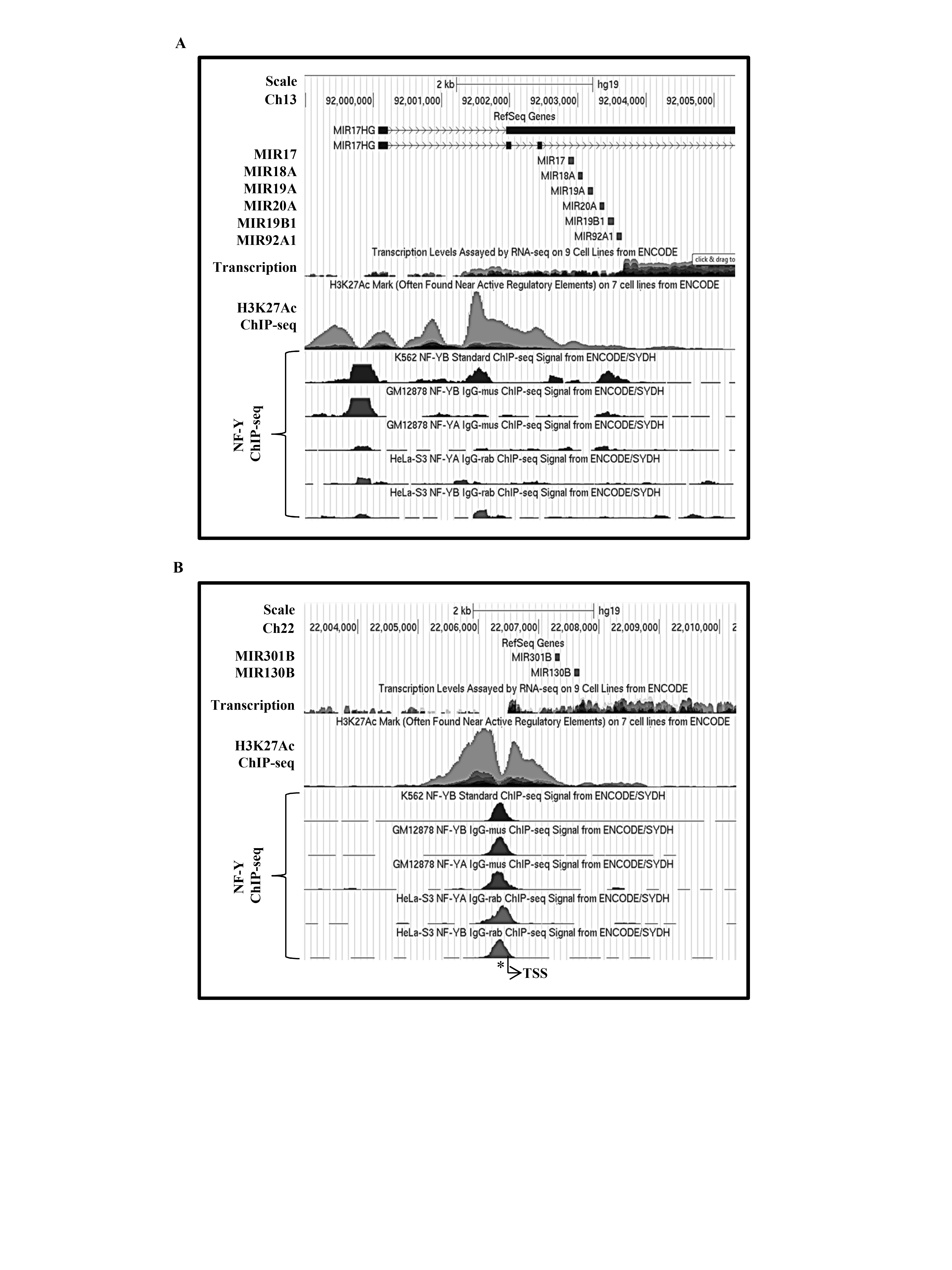

Supplement: S2 Fig — Screen-shot of UCSC Genome Browser at (A) miR-17-92a cluster genomic locus (B) miR-301b/130b genomic locus shows the position of mature miRNAs, transcription (RNA-seq data) and H3K27Ac ChIP-seq data (a marker of active promoter) from 9 cell lines (GM12878 (LCL), h1-hESC, HeLa-S3, HepG2, HSMM, HUVEC, K562, NHEK and NHLF), NF-Y ChIP-seq data from 3 cell lines. Arrow represent the proposed transcription start sites and the asterisk shows the position of primers used on our ChIP experiments shown on Fig 3. (TIF) [file pone.0153658.s008.tif]
